# Prophylactic Cauterisation in Rabies

**Published:** 1899-04-08

**Authors:** 


### PROPHYLACTIC CAUTERISATION IN RABIES.

At a recent meeting of the New York Pathological Society Dr. F. Cabot[1] described the results of a series of experiments which he had made with guinea-pigs to determine the effects of different forms of cauterisation on wounds after an interval of 24 hours from the time of infection with rabies. He said that it had been commonly believed that unless a cautery were applied within an hour after infection by a suspected animal it would be useless, and the result of this belief had been that physicians applying the cautery after a longer interval than this were apt to use it to a large extent as a matter of form, and thus to apply it in an ineffectual manner. Dr. Cabot's experiments may be shortly described as follows: A portion of the medulla taken from a rabbit dead from laboratory rabies was beaten into an emulsion with five times the quantity of sterile water. Of this emulsion one cubic centimetre was injected with a hypodermic needle into the outer and upper part of the thigh of a guinea-pig, in the region of the sciatic nerve. This injected virus was left undisturbed for 24 hours, at the end of which time an incision half an inch long was made over the seat of puncture, exposing the nerve. The tissue of the wound surrounding the puncture was then carefully swabbed out and the cautery was applied, the animal of course being under chloroform. In the first series of experiments, out of 60 animals injected, 34 were cauterised with nitric acid, with the result that 91 per cent. of them lived, while only 15 per cent. lived of the remainder which were not cauterised. In the second series, out of 59 animals injected, 44 were cauterised by the actual cautery. Of these 70 per cent. lived, while of the remainder only 11 per cent. lived. In the third series, out of 45 animals injected, 37 were cauterised by nitrate of silver, and of these 55 per cent. lived, while of the remainder 16 per cent. lived. In the fourth series, out of 49 animals injected, in 26 cases the wound was opened at the end of twenty-four hours and swabbed out with dry cotton wool. Of these 31 per cent. lived, while of the remainder, which were not touched, only 16 per cent. lived. These results are clearly very different from those obtained in similar experiments made upon wounds infected with septic matter. In such cases the materies morbi, being absorbed by way of the blood, is soon placed beyond the reach of any antiseptic proceedings. The poison of rabies, however, seems to travel towards the nervous centres by way of the wounded nerves of the injured part, and it is presumed that its progress along the nervous tissue, at first at any rate, is but slow. So far as they go these experiments tend to support the older clinical observations as to the efficacy of cauterisation, with this difference, however, that they strongly emphasize the advantage of a really strong, destructive, and widely-spreading caustic like nitric acid. It is impossible also not to see that, should these experiments receive confirmation, and we need hardly say that all experiments should be confirmed, it will become a very serious question, when a patient is bitten by a rabid animal on an easily removable part, such as a finger, whether amputation should not be at once performed, for if absorption is so slow that caustics can do good even at the end of 24 hours, amputation within the same period should be even more efficacious.

[1] Med. News, Mar. 18.

